# Level of Job Burnout among Midwives Working in Labour Rooms in Barcelona Region: A Cross-Sectional Study

**DOI:** 10.30476/ijcbnm.2021.88038.1504

**Published:** 2021-07

**Authors:** Pablo Rodríguez Coll, Rosa Cabedo Ferreiro, Roser Palau Costafreda, Laia Cantó Codina, Sergio García Perdomo, Noemí Obregón Gutiérrez, Ramón Escuriet Peiró

**Affiliations:** 1 Ghenders Research Group, School of Health Sciences Blanquerna, University Ramon Llull, Padilla, Barcelona, Spain; 2 Research Department in the Midwives’ Section of the Barcelona Nursing College (COIB), Barcelona, Spain

**Keywords:** Burnout, Delivery room, Hospital, Midwifery, Survey

## Abstract

**Background:**

Nowadays, burnout syndrome (BS) symptoms appear to have increased in healthcare workers, specifically midwives, but there are no studies on burnout among midwives in Catalonia. The present study aimed to assess and describe the prevalence of BS in midwives working in labour rooms.

**Methods:**

A cross-sectional descriptive study was conducted on 122 midwives working in 24 maternity hospitals in the Barcelona (region) which were selected using purposive sampling from January to March 2017. Data were collected using two questionnaires (demographic information, job burnout using Spanish Burnout Inventory with 20 items and four subscales). Data analysis was performed using SPSS software version 21 and Chi-Square, U Mann-Whitney, and Kruskall-Wallis. P<0.05 was considered statistically significant.

**Results:**

None of the participants obtained a critical level of BS. 37 (30.33%) participants scored medium burnout and 47 (38.52%) recorded low burnout. Statistically, work stress (P=0.01), marital status (P=0.006), attendance of more than three women per shift (P=0.001), the number of children (P=0.01), parity (P=0.005), health status (P=0.04), and being on sick leave over last year (P=0.04) were significantly correlated with medium-high levels of burnout. Burnout scores were higher in midwives having a life partner and those without children

**Conclusion:**

Following the results, no participant obtained a critical level of BS; about one-third of them scored medium-high burnout. However, specific interventions are suggested to be conducted to maintain the midwives’ motivation and prevent burnout development.

## INTRODUCTION

Burnout syndrome (BS) was first described in 1974 when Herbert Freudenberg observed how volunteers that worked in a drug addict’s clinic for a year developed symptoms such as energy loss, exhaustion, anxiety, and depression, or even became aggressive or demotivated. ^1^ Years later, BS was defined by Maslach as an inadequate response to chronic emotional stress characterised by three factors: emotional exhaustion, depersonalisation and reduced personal accomplishment. ^2 , 3^

The methods used to diagnose BS are ‘Maslach Burnout Inventory’ ^4^ or Copenhaguen Burnout Inventory, ^5^ some worldwide’s translated instruments that served as a precedent for subsequent investigations, Gil-Monte’s ‘Cuestionario para la Evaluación del Síndrome de Quemarse por el Trabajo’ ^6^ or the Spanish Burnout Inventory (SBI), a 20-item questionnaire specifically designed to evaluate BS among health professionals, teachers and public administration employees. ^7^

Regarding the healthcare system and specifically obstetric departments, the majority of the public hospitals in the National Health System have implemented a new model of care called “birth humanization” focused on women’s and their families’ needs. In this context, Midwives have a role in facilitating participation in the decision-making process and promote women’s autonomy and support their decisions, as specified in the World Health Organization (WHO) guidelines. ^8^ Thus, this model of care implies a more individualized and comprehensive care which can contribute to an increase in the midwives’ workload in a work environment where midwives probably have to look after multiple women during their shift; in consequence, it enhances the possibility of suffering from BS. ^9^ Furthermore, in countries like Spain, delayed motherhood has caused an increasing trend in the rate of women with high-risk pregnancies. ^10^ This implies in the exacerbation of maternal and neonatal results, due to the age-related risks. ^11^ All these factors, together with the current shortfall ratio of midwives, could lead to consequences such as job dissatisfaction, absenteeism, presenteeism, post-abandonment, depression, anxiety, insomnia, increase of occupational accidents, intention to leave one’s job, or higher predisposition to interpersonal conflicts among colleagues. ^12 , 13^ As to the potential factors that could provoke BS, some authors have reported excessive workload, unmanageable work schedules, inadequate staffing, low remuneration, and work environment among others. ^14 , 15^

The importance of the BS is such that last year the WHO has denominated it a work-related illness that worsens people’s physical and mental health. ^16^ Thus, health institutions are focusing their efforts on preventing the negative consequences of BS previously commented and creating a healthier work environment. ^17^

The most recent data from the Spanish National Institute of Statistics showed a 59% rate of BS prevalence among Spanish healthcare workers. ^18^ Moreover, the latter study carried out in Spain in 2019 has reported that 43.67% of nursing staff have burnout syndrome, ^19^ and some other investigations have shown a burnout prevalence from 18% to 33% among Spanish nurses and emergency healthcare workers. ^20 , 21^ Studies in other countries showed a great difference of BS levels among midwives, reaching up to 65% in Australian midwives, in contrast to 19.10% in Norwegian or 39% in Swedish ones. ^22 - 24^ In Spain, one of the latest studies showed a prevalence of 4.40% in midwives working in labour wards and 11.80% with high possibilities to develop BS. ^25^ So far, there are no studies on burnout among midwives in Catalonia.

The present study aims to describe the prevalence of BS among midwives that work in the labour rooms of 24 public maternity hospitals in Barcelona (Spain), identifying sociodemographic and work characteristics associated with this syndrome. 

## MATERIALS AND METHODS

This is a cross-sectional, descriptive study on a purposive sample of 122 midwives during January to March 2017 in Barcelona region. It was carried out using purposive sampling taking into account the study population registered in the Barcelona Nursing College in 2019. ^26^ The study population consisted of 260 midwives working in the 24 public hospitals within the Catalan Health System in the province of Barcelona (Spain). A total of 122 subjects randomly selected was sufficient with a 95% confidence and a desired precision of 0.1; the sample size was estimated around 50%. We anticipated a replacement rate of 25%. The sample size was calculated using the formula: 

$n=\frac{\left(Z2\times P\times (\mathrm{1-P})\right)}{e2}$

where Z is the value from standard normal distribution corresponding to desired confidence level (Z=1.96 for 95% CI), P is the expected true proportion, e is the desired precision (half desired CI width), and drop-out rate n*(1/1-Estimated Replacement Rate). ^27^

The data were collected among qualified midwives from all the 24 public maternity hospitals in the Barcelona province (Spain) and in agreement with the Catalan Healthcare Service. Inclusion criteria for this study were being a midwife working on labour rooms of public hospitals within the Catalan Health System in the province of Barcelona; being able to understand Spanish, and having basic computer knowledge to answer an online survey. Midwives working in community health centres, postnatal wards, or private hospitals were excluded.

The purposive sample was recruited throughout a mailing list, obtained from the Barcelona Nursing College, which was sent to every midwife supervisor or coordinator, inviting the midwives that met inclusion criteria to participate. The email explained the nature of the study and contained the link to the online survey, which took only 15 min to answer. The confidentiality, anonymity, and codification of the data for the study were maintained throughout the application of Survey Monkey. The participants should have specified their college number identification to have access to the survey and then they must have accepted the ethical conditions and given their consent before accessing the online questionnaires. Only the principal investigator and collaborators had access to the database. The recruitment was conducted for three months for every public hospital with a labour ward in Barcelona province. The study was reviewed and approved by the ethics committee of the Vall D’Hebron Barcelona Hospital Campus. The identification number is PR (AG) 25/2018.

All analyses were processed using SPSS Statistics, version 21 for Windows. Firstly, descriptive statistics (Mean, frequencies, standard deviation and percentages) were used to describe the midwives’ sociodemographic and job characteristics. Chi-Square test was used for testing the association between categorical variables (country, age, marital status, and years of experience) and BS scores. Secondly, we conducted inferential statistics analysis; U Mann-Whitney and Kruskall-Wallis were used to compare the medium-high BS level and sociodemographic and job data. P<0.05 was considered statistically significant.

Burnout syndrome levels were measured using the SBI, which is a 20-item self- report questionnaire. ^6 , 7^ Each item is rated on a five-point Likert scale from 0 “never” to 4 “every day”. All items are organized into four subscales: Enthusiasm toward the job (ETJ) (5 items, range=0-20), Psychological Exhaustion (PE) (4 items, range=0-16), Indolence (IN) (6 items, range=0-24), and Guilt (GU) (5 items, range=0-20). The first dimension is positive, while the other three are negative. ETJ means that professionals see their job as a source of personal accomplishment; PE refers to both physical and emotional exhaustion. Low scores on Enthusiasm toward the job, together with high scores on Psychological Exhaustion and IN, as well as on GU, indicate high levels of burnout. Cronbach’s alpha value for the ETJ subscale was 0.84, for the PE subscale 0.85, for the IN subscale 0.76, and for the GU subscale 0.80. Construct validity of the four-dimensional structure of the SBI was convincingly demonstrated by confirmatory factor analysis. The SBI had sufficient validity and reliability with good confirmatory factor analysis of the model (GFI=0.96, RMSEA=0.052, CFI=0.94 and NNFI=0.93). All the associations between the dimensions of the SBI were statistically significant, and all the factorial loadings were statistically significant for P<0.001. The whole instrument had an appropriate Cronbach alpha higher than 0.75. ^7^ Despite using the online version of the SBI questionnaire, the questionnaire maintained the validated psychometric properties previously reported in references ^6 , 7^ since the researchers did not change anything from the original. It is necessary to include every dimension score separately and then apply the following formula “20 - ETJ score + PE score + IN score/15” to obtain a total BS score. GU score is not necessary to obtain a total BS score. Afterwards, the total score of each dimension has to be divided by the number of items by which it is composed. E.g. ETJ score is 14/5 (since this dimension contains five items). With the two-decimal number obtained, called Direct Punctuation, it is necessary to review the scale for the corresponding percentile to which it belongs. The results interpretations are as follows: critical level (BS percentile >89), high level (BS percentile 67 to 89), medium level (BS percentile 34 to 66), low level (BS percentile 11 to 33), and very low level (BS percentile <11). ^6^

Demographic data collected included gender, age, marital status, parity, number of children and job data such as years of experience, workload or work shift among others. Respondents also indicated their health status and whether they had a stressful situation related to their issues or work.

## RESULTS

Of the 260 midwives, 242 were eligible after exclusion of 14 participants (moved to another region, wrong email or unknown causes) and 4 participants who were retired. A total of 122 midwives answered the questionnaire, representing a 50.41% response rate. The majority of participants were women 118 (96.70%). The age range of the participants was 25–64 years with a mean of 40±10.88. The midwives’ sociodemographic and job characteristics are shown in Table 1.

**Table1:** Midwives’ sociodemographic and job characteristics (N=122)

Variable	N (%)
**Marital status**	
Unmarried	34 (27.90)
Having a life partner	82 (67.20)
Separated/divorced	6 (4.90)
**Hospital Classification**	
1st-level hospital (50-250 beds)	2 (1.64)
2nd-level hospital (200-800 beds)	56 (45.90)
3rd-level hospital (300-1500 beds)	64 (52.46)
**Years of experience**	
>10 years	67 (55)
6-10 years	22 (18)
1–5 years	26 (21.30)
<1 year	7 (5.70)
**Work Shift**	
Day shift (without nights)	56 (45.90)
Night shift (without days)	34 (27.90)
Rotating shift (day and night)	32 (26.20)
**Workload**	
1-3 women in labour per shift	79 (64.75)
4-5 women in labour per shift	32 (26.23)
>5 women in labour per shift	11 (9.02)
**Considered Health Status**	
Very good	71 (58.20)
Good	25 (20.50)
Regular	26 (21.30)
Bad	0 (0)
**Sick leave last year**	
Yes	90 (73.80)
No	32 (26.20)
**Work and stress**	
Work causes participants high stress	15 (12.30)
Work causes participants moderate stress	69 (56.60)
Work causes participants any or little stress	38 (31.10)
**Familiar and/or personal situation and stress**	
Familiar and/or personal situation could cause participants stress	76 (62.30)
Having a familiar and/or personal situation with stress	46 (37.70)

The mean score of ETJ subscale was 3.10±0.85, and those of the other subscales were PE (1.84±1.07), IN (1.04±0.74) and GU (0.82±0.79). Regarding ETJ, only 31 (25.41%) participants obtained high levels in this subscale, 46 (37.71%) scored medium level scores, and 45 (36.88%) scored low levels of ETJ. Furthermore, 65 (53.28%) participants scored a medium level of PE, and 68 (56.66%) obtained medium level scores of IN. Moreover, 62 (50.82%) participants obtained medium level scores of GU. No one was in the critical level of BS. BS prevalence is shown in Table 2.

**Table2:** Prevalence of burnout category in midwives (N=122)

Level	N (%)
Critical (Burnout percentile >89)	0
High (Burnout percentile 67 to 89)	1 (0.82)
Medium (Burnout percentile 34 to 66)	37 (30.33)
Low (Burnout percentile 11 to 33)	47 (38.52)
Very Low (Burnout percentile <11)	37 (30.33)

Burnout scores were higher in midwives having a life partner (P=0.006) and those with no children (P=0.007). The comparison of burnout scores higher than 45% according to sociodemographic and job variables is shown in Table 3 and the association of medium-high burnout level with sociodemographic and job data is shown in Table 4. 

**Table3:** Comparison of burnout scores higher than 45% according to sociodemographic and job variables

Variables name	Burnout scores higher than 45% N (%)	P value
**Age**		0.91*
25 to 36 years old	44 (36.07)		
36 to 64 years old	78 (63.93)		
**Marital Status**		0.006*
Having a life partner	92 (75.56)		
Unmarried	27 (22.22)		
**Years of experience**		0.32**
1-5 years of experience	45 (37.04)		
6-10 years of experience	22 (18.18)		
>10 years of experience	20 (16.67)		
**Offspring**		0.007*
With no children	35 (28.57)		
With one or more than one child	15 (12.12)		

* U Mann-Whitney,

** Kruskal-Wallis

**Table4:** Association between medium-high burnout level and sociodemographic and job data

Sociodemographic and job data	Variables	Medium-high burnout level mean±SD^a^	P value
**Considering work stress**	Yes	19±10	0.010*
	No	42±13		
**Marital status**	Unmarried	16±2	0.006**
	Having a life partner	41.5±22.50	
	Separated/divorced	3.5±1.50	
**Attending more than three women per shift**	Yes	18±0	0.001*
	No	43±23	
**Third level hospital**	Yes	30.5±8.50	0.265*
	No	30±14	
**More than ten years of work experience**	Yes	27.5±8.50	0.463*
	No	33.5±14.50	
**Working on a night shift**	Yes	27±10	0.943*
	No	34±13	
**Number of children**	0-2	27±10	0.012*
	3-4	6±4	
**Hospital level**	First level	5±1	0.563**
	Second level	26±80	
	Third level	30±14	
**Working shift**	Day shift (without nights)	27±10	0.516**
	Night shift (without days)	17±80	
	Rotating shift (day and night)	17±50	
**Parity**	Between 37 and 41 gestational weeks	28±3.50	0.005*
	More than 41 gestational weeks	32.5±19.50	
**Staff ratio**	Considered enough	50.5±18.50	0.882*
	Considered not enough	10±4	
**Contract type**	Staff with a permanent position	46.5±19.50	0.366*
	Eventual staff	14.5±3.50	
**Health status**	Regular	10±30	0.047**
	Good	37±19	
	Very good	14.±1	
**Being in sick leave**	Yes	10±3	0.048*
	No	50±20	

a Standard Deviation,

* U Mann-Whitney,

** Kruskal-Wallis

High ETJ subscale scores showed a statistically significant association with stress (P=0.004). Furthermore, there was a statistically significant association between high guilty subscale scores and more than ten years of work experience (P=0.02). Finally, there was a statistically significant association between high IN subscale scores and more than ten years of work experience (P=0.01), labour stress (P<0.001), contract type (P=0.01), and staff ratio (P=0.01).

## DISCUSSION

Although our findings showed that no participant obtained a critical level of BS, about one-third of them scored medium-high burnout, which indicates a high risk of developing burnout. In addition to the results obtained in other studies, having a life partner and having no children constitute the main related factors to develop BS. ^28 - 30^ Although almost sixty four percent of midwives aged between 36 and 64 years old and thirty seven percent of those with less than 5 years of work experience scored medium-high burnout, there were no statistically significant association among them. Even though it could seem contradictory that the less experienced midwives and the oldest ones have higher levels of burnout, it could be explained due to the stress and the anxiety in less experienced midwives recently graduated who feel job insecurities and the fatigue and continued anxiety perceived in senior midwives, in line with some other authors’ findings. ^31 , 32^ This highlights an upcoming concern: those little experienced midwives recorded some of the highest burnout scores, so there is an important scope for change across the labour wards where proactive support needs to be offered to younger, recently qualified midwives, to help sustain their emotional wellbeing. A possible solution could be the one implemented in the UK model, providing regular and specific work-related education and organizing rotating shifts combining senior midwives and recently qualified ones to reduce stress level to those midwives with less experience. ^33^ Another important aspect to be taken into consideration and could also explain the results previously commented, is that Spanish midwives go through nursing education before access to midwifery education, being at least 24 years old when they qualify as midwives. However, some nurses have access to the midwives’ speciality when they have already worked as nurses for many years. 

In line with the results obtained in other investigations, stressors such as looking after more than three women per shift, being on sick leave during the previous year and considering work stress have a statistically significant association with medium-high BS levels. ^34 , 35^ Personal factors and working conditions should be taken into account when assessing burnout risk profiles of the midwives, and measures should be taken to create a healthier work environment in hospitals, such as train midwives to recognise burnout symptoms and offer them support when needed. ^36 , 37^ On the contrary, almost two-thirds of the participants recorded low or very low burnout. 

Even though we could not find a cross-national study of midwives in Catalonia, our results obtained higher scores in ETJ subscale than those observed in health professionals from Italy. ^38^ However, the rest of the subscales showed similar results.

Another important aspect to be taken into account in our study is that there was a statistically significant association among high IN subscales and some factors such as more than ten years of work experience, labour stress, contract type, and staff ratio. These results have been obtained in similar settings and among midwives’ staff in a recent study where some recommendations were proposed to avoid burnout syndrome. These include having a good midwifery rate per patient, providing appropriate quality of care, i.e. changing the midwifery staffing standards at hospitals, reorganizing services so that midwives can practice to their full scope, and providing continuity of care. ^39^

The main relevance of this study is that Barcelona midwives do not reach any critical level of BS. However, there are BS important levels in midwives who have a life partner and no children. The Spanish Burnout Inventory provides the researchers and practitioners with an expanded conceptualization of the burnout syndrome, which can facilitate the diagnosis and treatment of this syndrome. ^40^

There are some potential limitations in this study that should be mentioned; limiting the study to midwives working in a hospital setting only may have been a bias regarding the inclusion criteria since other healthcare professionals as community midwives or midwives working in private labour rooms were excluded. 

It should be remembered that the data were collected three years ago, and the changes which occurred in the health field due to COVID-19 could have affected the midwives’ outlook. Therefore, it would be necessary to repeat the study in future research, expanding a sample with a population from different Spanish regions and similar hospital conditions or pregnancy services. It would also be interesting to study other types of variables that could lead to burnout syndrome. The variables related to burnout that appear in the scientific literature have been taken into account. In this study, the association between BS and midwives’ relationship with peers and other obstetric professionals has not been considered, as well as multiple employment types, as it was not the main purpose of the study. 

On the other hand, this study has several strengths; it is the first study describing burnout prevalence in midwives working in Catalonia; no studies have been conducted using SBI in the midwifery population.

Specific sociodemographic and job characteristics, like having a life partner or having no children have been associated with burnout, which might be useful to take actions to improve the healthcare system and focus on supporting newly qualified midwives during their first years of practice. 

## CONCLUSION

We described the prevalence of BS in midwives working on the labour wards in Barcelona public Hospitals. The results showed that midwives having a life partner and those with no children obtained higher levels of BS. Institutions and policymakers should better understand the sociodemographic factors and job stressors within the midwifery population to maintain a healthy work environment and keep midwives motivated to improve the quality of care in obstetric services. 

Further investigation is needed in the areas that could be improved, such as care load, human resources, educational support, and work shifts, among others, to propose possible strategies to avoid and prevent the appearance of the BS and, therefore, improve the quality of care. In future research, it would be interesting to conduct a case-control study in other areas of Spain and different pregnancy care settings (community, labour ward, postnatal wards, or even private institutions) to know the other risks (odds ratio) of the related factors identified with BS.
